# Cancer Predisposition Syndromes and Medulloblastoma in the Molecular Era

**DOI:** 10.3389/fonc.2020.566822

**Published:** 2020-10-29

**Authors:** Roberto Carta, Giada Del Baldo, Evelina Miele, Agnese Po, Zein Mersini Besharat, Francesca Nazio, Giovanna Stefania Colafati, Eleonora Piccirilli, Emanuele Agolini, Martina Rinelli, Mariachiara Lodi, Antonella Cacchione, Andrea Carai, Luigi Boccuto, Elisabetta Ferretti, Franco Locatelli, Angela Mastronuzzi

**Affiliations:** ^1^ Department of Hematology/Oncology, Cell and Gene Therapy, Bambino Gesù Children's Hospital, IRCCS, Rome, Italy; ^2^ Department of Molecular Medicine, Sapienza University of Rome, Rome, Italy; ^3^ Department of Experimental Medicine, Sapienza University of Rome, Rome, Italy; ^4^ Oncological Neuroradiology Unit, Imaging Department, Bambino Gesù Children's Hospital, IRCCS, Rome, Italy; ^5^ Department of Neuroscience, Imaging and Clinical Science, University “G.d’Annunzio” of Chieti, Chieti, Italy; ^6^ Laboratory of Medical Genetics, Bambino Gesù Children's Hospital, IRCCS, Rome, Italy; ^7^ Neurosurgery Unit, Department of Neurological and Psychiatric Sciences, IRCCS Bambino Gesù Children’s Hospital, Rome, Italy; ^8^ JC Self Research Institute, Greenwood Genetic Center, Greenwood, SC, United States; ^9^ School of Nursing, College of Behavioral, Social and Health Science, Clemson University, Clemson, SC, United States; ^10^ Department of Maternal, Infantile, and Urological Sciences, University of Rome La Sapienza, Rome, Italy

**Keywords:** pediatric brain tumors, cancer predisposition, hereditary neoplastic syndromes, cancer syndromes, medulloblastoma, cancer genes

## Abstract

Medulloblastoma is the most common malignant brain tumor in children. In addition to sporadic cases, medulloblastoma may occur in association with cancer predisposition syndromes. This review aims to provide a complete description of inherited cancer syndromes associated with medulloblastoma. We examine their epidemiological, clinical, genetic, and diagnostic features and therapeutic approaches, including their correlation with medulloblastoma. Furthermore, according to the most recent molecular advances, we describe the association between the various molecular subgroups of medulloblastoma and each cancer predisposition syndrome. Knowledge of the aforementioned conditions can guide pediatric oncologists in performing adequate cancer surveillance. This will allow clinicians to promptly diagnose and treat medulloblastoma in syndromic children, forming a team with all specialists necessary for the correct management of the other various manifestations/symptoms related to the inherited cancer syndromes.

## Introduction

Medulloblastoma (MB) is the most frequent malignant tumor of the central nervous system (CNS) in childhood, representing 15–20% of all CNS neoplasms (1). It mainly affects the pediatric age with a 10-fold higher frequency than in adults (2). Children are diagnosed generally between 2 and 8 years old (median of 6 years old), with 50% of cases occurring in children under 5 years old and with a male/female ratio of 2:1 (3).

Clinical manifestations are initially related to intracranial hypertension and to the tumor’s mass effect in the posterior fossa, including headaches, nausea, vomiting, ataxia, other motor deficits, and visual impairment. MB diagnosis is suspected based on neuroimaging of the brain and spine. Disease staging is established on magnetic resonance imaging (MRI) and cerebrospinal fluid (CSF) cytology (4), with about 35% of cases being metastatic at diagnosis (5).

Histological classification of MB distinguishes four variants: classic (68–80%); desmoplastic/nodular (7%), with a more favorable prognosis in children under 5 years old; MB with extensive nodularity (3%), generally found in young patients and sometimes associated with nevoid basal cell carcinoma syndrome; and large cell/anaplastic (10–22%), characterized by a more aggressive clinical behavior (6).

Treatment of MB is based on surgical resection, chemotherapy, and cranio-spinal irradiation (CSI). Due to the severe adverse effects of CSI, such as neurocognitive disability, endocrine dysfunction, impaired growth, infertility, and increased risk of secondary malignancies, great effort has been dedicated to reduce, differ, or omit radiation therapy, especially in children <3–5 years of age.

Among genetic defects, MYC amplification is the most recurrent and is associated with a worse prognosis (7–9).

A risk stratification based on histopathological subtype, age at diagnosis, staging, residual disease, MYC status, and molecular subgrouping allows a distinction of low-, average-, and high-risk patients (10). For low- and average-risk patients (characterized by age over three years old, absence of metastatic and/or residual disease, histotype other than anaplastic, absence of MYC amplification and/or TP53 mutations), 5-year overall survival (OS) is between 75% to over 90% (11–14), while high-risk patients show 5-year OS around 50–75% (11, 15–19).

More recently, four molecular MB subgroups have been identified and included in the 2016 WHO Classification of Tumors of the Central Nervous System (20): MB_WNT_, MB_SHH_, Group 3, and Group 4 (21). Molecular subgrouping reflects developmental aspects of the tumors’ cell of origin and has been shown to have prognostic significance.

Cancer predisposition syndromes’ importance has increasingly been recognized in pediatric neuro-oncology. According to Waszak et al. germline mutations in cancer predisposition genes account for about 5–6% of medulloblastoma diagnoses (22). Constitutional genetic defects are expected to result in deregulation of specific molecular pathways, leading to tumor development. Despite the significant amount of previous knowledge on inherited conditions predisposing to MB and the extensive molecular characterization of these tumors, limited attention has been given in the literature to their interconnection.

The main purpose of this review is to describe the association of cancer predisposition syndromes with MB molecular subgroups, including epidemiological, clinical, genetic, diagnostic, and therapeutic implications.

## Methods

**Figure 1:**
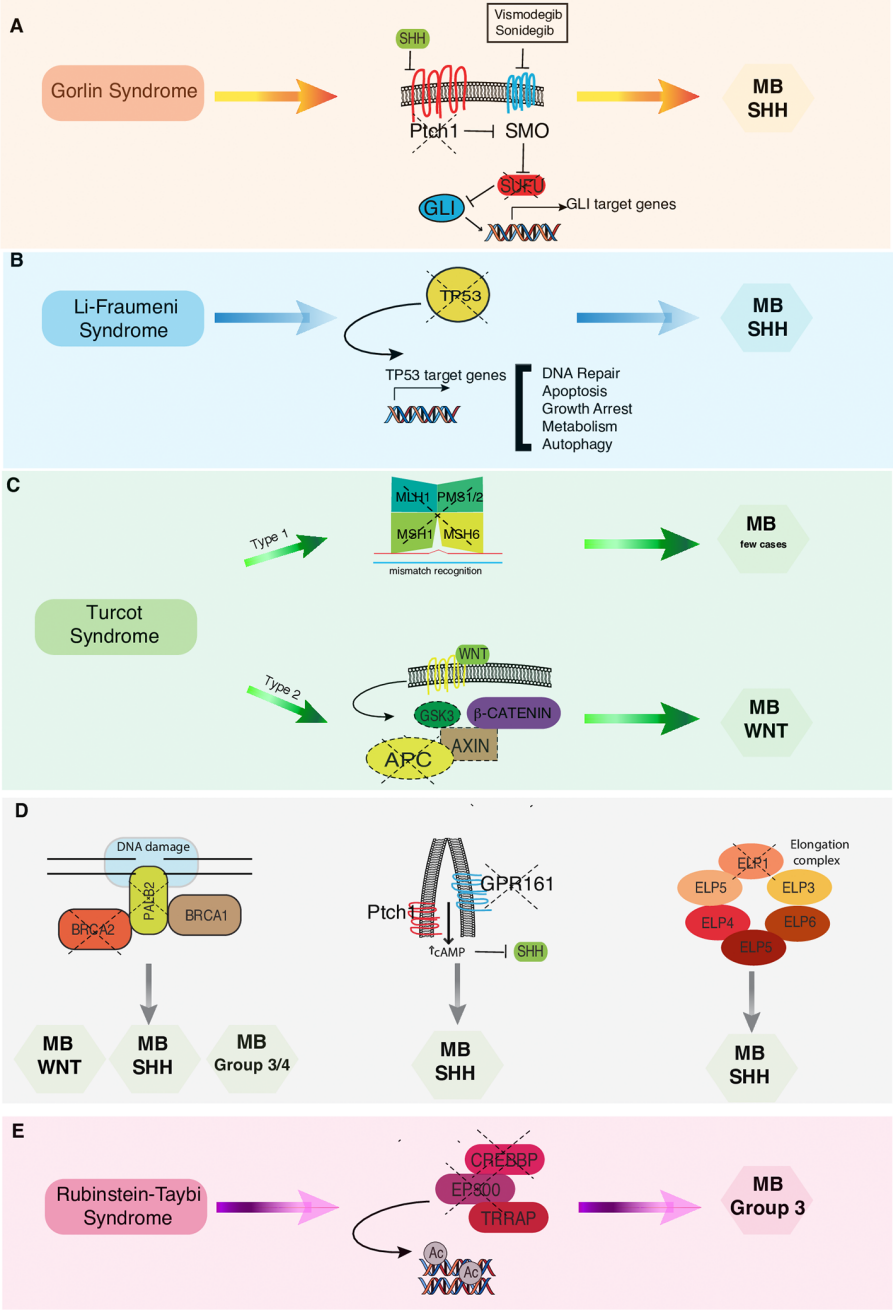
Correlations between cancer predisposition syndromes and MB subtypes. **(A)** In Gorlin syndrome both PTCH1 and SUFU mutations have been associated to MB-SHH subgroup. Vismodegib and Sonidegib are selective antagonists of the transmembrane activator Smoothened (SMO). **(B)** In Li-Fraumeni syndrome los of TP53 finctions results in increased risk of developing MB-SHH subtype. **(C)** In Turcot syndrome, twotypes have been distinguished: Type 1 genetically related to the mutationof the mismatch repair genes and Type 2 related to APC mutation that are more commonly associated with MB-WNT subtype. **(D)** Pathogenic germline mutations in BRCA2, PALB2, GPR161, and ELP genes have been recently associated to an increased risk of developing different MB subtypes. **(E)** In Rubenstein-Taybi syndrome mutations in CREEBP and EP300 genes predispose to MB Group 3 onset.

The authors conducted a literature search describing the issue of CNS tumors and cancer predisposition syndromes. Research studies were selected based on research topics (“cancer predisposition syndrome,” “brain tumor genetics,” “brain tumor cancer predisposition syndrome,” “medulloblastoma predisposition syndromes,” “medulloblastoma in childhood”) found in PubMed considering the last 10 years until April 2020. These studies were classified according to their relevance. In the selected studies the data were carefully evaluated, and they are described in detail and discussed in the following sections. The association between the different cancer predisposition syndromes described below and the related molecular subgroups of MB is summarized in **Figure 1**. The main cancer predisposition syndromes associated to pediatric MB and their related molecular, pathological, clinical, and prognostic features are summarized in **Table 1**.

**Table 1:** Cancer predisposition syndromes associated to pediatric medulloblastoma and their related molecular, pathological, clinical, and prognostic features.

Predisposition genes	Cancer syndrome	MB prevalence (%)	MB median age at diagnosis (years)	Molecular subgroup	MB histologic subtype	Clinical features	5 year-OS(%)	References
*PTCH1*	Gorlin	<2–4.5	2	SHH	Desmoplastic/nodular with extensive nodularity	Palmar or plantar pits, odontogenic keratocysts, basal cell carcinomas	85^*^	Waszak et al. (23)
*SUFU*	Gorlin	2–33	2	SHH	Desmoplastic/nodular with extensive nodularity	Palmar or plantar pits, odontogenic keratocysts, basal cell carcinomas	85^*^	Waszak et al. (23); Smith et al (24)
*TP53*	Li Fraumeni	1	9.8	SHH WNT	LCA, Classic	Soft tissue sarcomas, osteosarcomas, glioblastomas/astrocytomas, choroid plexus carcinomas, breast cancers	27	Waszak et al. (23); Zhukova et al. (25)
*MLH1, MSH2, MSH6, PMS1, PMS2*	Turcot type1	unknown	unknown	unknown	unknown	Café-au-lait spots	unknown	
*APC*	Turcot type2	1	9.2	WNT SHH (rarely)	Classic	Gastrointestinal symptoms (diarrhea, constipation), neurological symptoms (headache, vomiting, visual and/or hearing and/or sensorimotor deficits)	80-100	Waszak et al. (23); Surun et al. (26)
*BRCA2*	unknown	1	5.7	SHH WNT SHH	Classic, desmoplastic/nodular, LCA, with extensive nodularity Classic	unknown Fanconi Anemia phenotype (biallelic mutations)	25**;100***	Waszak et al. (23) Present report Present report
*PALB2*	unknown	<1		SHH Group3 Group 4	unknown	unknown	75	Waszak et al. (23)
*GPR161*	unknown	3.4^****^	unknown	SHH	unknown	unknown	unknown	Tischkowitz et al. (27)
*ELP1*	unknown	unknown	6.3	SHH	Desmoplastic/nodular	unknown	92	Hwang et al. (28)
*CREBBP; EP300*	Rubinstein-Taybi	0.05^*****^	unknown	Group3^*****^	unknown	Growth retardation, obesity, facial, skeletal and neurological anomalies, cognitive/psychiatric disorders, pilomatricomas	unknown	Carter et al. (29)

* cumulative PTCH1 and SUFU.

** compound heterozygous BRCA2.

*** heterozygous germline BRCA2.

**** referred to patients with MB_SHH_ subgroup.

***** limited data.

### Medulloblastoma Molecular Subgroups

Main features of MB subgroups are:

- Wingless (WNT) accounts for about 10% of diagnoses and is found mainly in girls with a peak between 10 and 12 years of age. The most common histological variant is classic. Approximately 85–90% of MB_WNT_ harbor somatic mutations in exon 3 of *Catenin beta 1 (CTNNB1)*, which causes stabilization and nuclear accumulation of β-catenin leading to uncontrolled activation of WNT signaling (23, 30). Patients with MB_WNT_ without *CTNNB1* mutations can harbor a mutant *APC* tumor suppressor gene, which is involved in the ubiquitination and consequently degradation of β-catenin (22). MB_WNT_ have a low tendency to metastasize and patients under 16 years of age have an excellent prognosis. Therefore, some ongoing clinical trials, PNET5 and SJMB12, are currently investigating de-escalation of therapy (19).
- Sonic hedgehog (SHH) accounts for about 30% of all MB diagnoses and has a bimodal distribution, with peaks in children <3 years of age and in young adults >16 years of age (21). This subgroup affects both sexes almost equally with a slight predominance in males among infants (31). The histological variant is frequently desmoplastic/nodular. MBs-_SHH_ harbor germline or somatic mutations in genes involved in SHH signaling pathway, leading to its constitutive activation, such as deletions or loss-of-function alterations in *Patched 1 (PTCH1)* (43% of patients) or *Suppressor of fused (SUFU)* (10%), activating mutations in *Smoothened (SMO)* (9%), amplification of *GLI1*/*GLI2* (9%) or *MYCN* (7%) (23, 32). More recently, four SHH subtypes have been identified (SHH_α_, SHH_β_, SHH_γ_, SHH_δ_) with distinct biological and clinical features (33). Older children with MB_SHH_ can harbor germline or somatic *Tumor Protein 53* *(TP53)* mutations, associated with a poor prognosis (25, 32).
- Group 3 accounts for about 25–28% of all MB diagnoses and is exclusively found in childhood, with a male sex predominance. It is associated with metastatic disease at diagnosis and with large cell/anaplastic histological variant. About 17% of Group 3 MBs harbor *MYC* amplification. Among MB subgroups, Group 3 is characterized by the poorest prognosis, especially in the presence of metastatic disease, isochromosome 17q, and *MYC* amplification (19).
- Group 4 is the most common MB molecular subgroup, accounting for about 35% of diagnoses. It is mostly found in males and more frequently associated to classic histological variant. It is characterized by an overall intermediate prognosis; however, a subset of patients with either chromosome 11 loss or 17 gain have an excellent prognosis (19).

## Gorlin Syndrome

Gorlin syndrome (GS) (OMIM #109400), also known as Gorlin-Goltz syndrome, or nevoid basal cell carcinoma syndrome (NBCCS), or basal cell nevus syndrome (BCNS), was first described by Gorlin and Goltz in 1960 (34). The incidence of GS reported is about 1 in 15.000 births (35) and is equal between males and females (36). The prevalence varies from 1:30,000 to 1:256,000 based on different reports (37–40). Prevalence data could be even greater since milder cases of GS could remain undiagnosed (41, 42).

### Clinical Phenotype

GS is characterized by the onset of multiple jaw keratocysts, most frequent in the second decade of life, and/or basal cell carcinomas (BCCs), generally starting from the third decade. Sixty percent of all patients have a recognizable phenotype. More than 100 features have been associated with GS, and the most representative are listed in **Table 2** (39, 40, 43).

**Table 2:** Principal clinical features associated with Gorlin Syndrome.

Clinical features	Description
**Macrocephaly**	Head circumference increases above 97th percentile until age 10 to 18 months and then maintains its centile
**Facies features**	Frontal bossing, coarse facial features, and facial milia in about 60% of individuals with *PTCH1* mutation; more subtle in individuals with *SUFU* mutation
**Jaw keratocysts**	Can arise early as from five years of age, with a peak in the teenage years; usually present with painless swellings and if untreated can lead to tooth disruption and jaw fracture
**Other congenital malformations**	Cleft lip/palate; polydactyly; skeletal anomalies (bifid ribs, wedge-shaped vertebrae, short 4th metacarpal); various eye anomalies (strabismus, hypertelorism, cataract, orbital cyst, microphthalmia, retinal epithelium alterations)
**Skin anomalies**	Pits in the palm of the hand
**Other anomalies**	* Ectopic calcifications, frequently in the falx cerebri in more than 90% of patients by age 20 years

### Genetic Basis

Heterozygous germline mutations leading to the aberrant activation of SHH signaling are involved in GS, most frequently *PTCH1*, followed by *SUFU*. *PTCH1* and *SUFU* mutations work at different levels by disabling SHH pathway signaling, which is normally active during brain development, thus promoting proliferation and inhibiting apoptosis (24, 44–47).

### Correlation With Medulloblastoma

In 1963 Herzberg and Wiskemann first described the association between GS and MB that has been also confirmed by various published studies (48).

In the first large population based study of GS, Evans et al. investigated the incidence of GS in 173 consecutive cases of MB in the North-West of England between 1954 and 1989; they observed a 5% incidence of GS in MB patients with less than 5 years of age, conversely, the incidence of MB in the GS population considered in this study was 3.6% (49). The mean age at MB diagnosis was 2 years in GS patients, earlier than that described in the general population with sporadic MB (38). The desmoplastic/nodular and the extensive nodularity subtypes of MB are the most frequently described (50, 51). The risk of MB in subjects with germline mutations of PTCH1 reported in a large series of 115 individuals with related GS-PTCH1 was <2%, while individuals with GS and SUFU germline mutations presented an approximately 20 times higher risk (33%) (24).

### Diagnosis

Many individuals with GS are only recognized in adulthood. However, there are clinical signs that could appear early and guide the diagnosis, such as the presence of odontogenic keratocysts in children <20 years of age, basal cell carcinomas in persons <20 years of age, palmar or plantar pits, lamellar calcification of the falx cerebri, and MB with desmoplastic histology in combination with other major or minor criteria (52). Current diagnostic criteria for GS are summarized in **Table 3**. Diagnosis can be made if 2 major or 1 major and 2 minor criteria are fulfilled (36).

**Table 3:** Current diagnostic criteria for Gorlin Syndrome.

**Major Criteria**	Multiple basal cell carcinomas (more than five in a lifetime) or basal cell carcinoma occurring at a young age (<30 years old)
	Jaw keratocysts
	Two or more palmar/plantar pits
	Lamellar calcifications of the falx cerebri or clear evidence of calcification in an individual younger than age of 20 years
	First degree relative with Gorlin Syndrome
**Minor Criteria**	Childhood medulloblastoma
	Lympho-mesenteric or pleural cysts
	Macrocephaly (>97th percentile)
	Cleft lip/palate
	Rib anomalies (bifid, splayed, extra ribs) or vertebral anomalies (bifid vertebrae)
	Ocular anomalies (cataract, developmental defects, pigmentary changes of the retinal epithelium)

### Cancer Surveillance

Surveillance protocols for individuals affected by GS have been proposed by several authors. As suggested in the consensus statement from the first international colloquium on GS, all individuals with GS should perform annually an assessment with a geneticist. A dermatological evaluation is also recommended annually until the first basal cell carcinoma is found, and then every 6 months. Baseline digital Panorex of jaw should be performed starting from the age of 3 years (or as soon as tolerated) and repeated annually before the detection of a first jaw cyst, and then every 6 months (until no jaw cyst for 2 years or until the age of 21).

A baseline echocardiographic evaluation is recommended to exclude cardiac fibromas; in females a pelvic ultrasound for fibromas is also recommended, starting from puberty.

A baseline spine film should be performed at age 1 or at time of diagnosis, and if a skeletal anomaly is found, it must be repeated every 6 months, or sooner if necessary. A routine developmental screening, including an assessment of vision, hearing, and speech, is recommended annually.

Annual brain MRI with contrast has been recommended until the age of 8 (52).

However, Smith and colleagues recently described the risk stratification of MB development between *PTCH1* and *SUFU* mutation carriers, recommending the performance of brain MRI only for patients carrying *SUFU* mutation (24).

Expert consensus recommendations for tumor surveillance of gene carrier and family members were proposed in 2016 based on a literature review and discussion in the AACR Childhood Cancer Predisposition Workshop held in Boston, Massachusetts, in October 2016 (see **Table 4**) (53).

**Table 4:** Gorlin Syndrome surveillance recommendations.

***PTCH1* mutation carriers**	Basal cell carcinoma screening annually by age 10, with increased frequency after first basal cell carcinoma observed
	Baseline echocardiogram in infancy, dental exams with jaw X-ray every 12 to 18 months beginning at age 8, and an ovarian ultrasound by age 18
	Low risk of medulloblastoma: no radiographic screening unless concerning neurologic exam, head circumference change, or other unusual signs or symptoms
	If medulloblastoma: radiation-sparing treatment given risk of radiation-induced skin cancers
***SUFU* mutation carriers**	Same as *PTCH1* mutation carriers, with the exception of no jaw X-rays, as keratocysts have not been described
	Additional medulloblastoma screening: consider every 4 month brain MRI through age 3 and then every 6 month brain MRI until the age of 5^a^. Radiation-sparing treatments are again recommended if a brain tumor should occur

a Data to support optimal frequency and timing of imaging are not currently available.

### Therapeutic Approaches

Vismodegib and Sonidegib are selective antagonists of the SHH pathway that act by binding to the transmembrane activator SMO, inhibiting the activation of the downstream SHH pathway.

Vismodegib is the first SHH pathway inhibitor approved by U.S. Food and Drug Administration (FDA) in 2012 and by European Medicines Agency in 2013 for the treatment of advanced or metastatic basal cell carcinomas (54, 55).

Sonidegib is approved by the FDA in adult patients for the treatment of locally advanced recurrent basal-cell carcinomas after radiation or surgery or for patients that cannot undergo surgery or radiotherapy (56).

A systemic review and meta-analysis about phase I and phase II Sonidegib and Vismodegib clinical trials highlighted that they are both well tolerated and with anti-tumor activity in MB_SHH_. The efficacy of Sonidegib was better than Vismodegib in pediatric MB_SHH_; however, this has been observed in 3 pediatric patients and further studies are needed for a reliable result (57).

Since SHH signaling has a crucial role during development, along with reports of younger patients treated with SMO inhibitors that show various growth plate complications, their use is not recommended in skeletally immature patients (58).

## Li-Fraumeni Syndrome

Li-Fraumeni Syndrome (LFS) (OMIM #151623) is one of the most aggressive cancer predisposition syndromes, first described in 1969 by Frederick Li and Joseph Fraumeni Jr (59). LFS is a rare autosomal dominantly inherited disorder caused by germline mutation of *TP53*, the “guardian of the genome” (60–62). Loss of p53 function in affected individuals is responsible for an increased risk of developing various solid and hematologic cancers (63). LFS has an estimated prevalence of 1 in 5,000 to 1 in 20,000 (64, 65). However, according to Andrade et al., prevalence estimates of the LFS could be higher (1 in 3,555–5,476), reflecting the complexity linked to a wide phenotype and a variable penetrance (66).

### Genetic Basis

*TP53* gene is located at chromosome 17p13.1 and is composed by 14 coding exons: 10 encode TP53 protein, one a non-coding exon, and three alternative exons (67). *TP53* acts as a tumor suppressor gene: in unstressed cells TP53 is unstable and, after exposure to genotoxic stressors, it accumulates and induces the expression of various target genes involved in the regulation of critical cellular processes (growth suppression, apoptosis, DNA repair). Various mechanisms have been proposed to explain how the mutated TP53 protein contributes to tumor formation, including loss of TP53 tumor suppressor function and consequently the dysregulation of its target genes, the “dominant negative” effect in which the mutated TP53 protein inhibits wild-type TP53 protein and the “gain-of-function effect” in which the altered TP53 protein acquires new oncogenic properties.

### Clinical Phenotype

Both children and adults affected by LFS have an increased risk of developing multiple primary tumors (68). The most frequent six “core” cancers, their relative prevalence estimates, and other less frequent types of tumor reported in LFS are summarized in **Table 5** (60, 69, 70). Considering all ages, the most frequent tumor reported in LFS families is breast cancer, with a median age at onset of 33 years in females (65, 70–73). Soft tissue sarcomas and osteosarcoma are the most common tumors in children and adolescents with LFS (65, 70, 74). The most common type of CNS tumors is glioblastoma/astrocytoma (65, 71). Choroid plexus carcinomas (CPC) are more tightly associated with LFS since 45–100% of children with CPC show a germline TP53 mutation (65, 75–78).

**Table 5:** Types of cancer associated with Li-Fraumeni Syndrome.

Cancer types in Li-Fraumeni Syndrome			Prevalence (%)
Most frequent six “core” cancers	Premenopausal Breast Cancer		27–31
	Soft Tissue Sarcomas		17–27
	Osteosarcoma		13.4–16
	CNS Tumors		9–14
	Adrenocortical Carcinoma		6–13
	Leukemia		2–4
Other less frequent cancer types	Myelodysplastic Syndrome Lymphoma Lung Laryngeal	Thyroid Gastrointestinal tract Kidney Testicular	Prostate Ovarian Skin Neuroblastoma

### Correlation With Medulloblastoma

Although MB has been described in families with LFS, its prevalence in *TP53* carriers is not well known (79). About 5–10% of MBs present *TP53* mutations; however, most of these are somatic and only 1% of MBs have been associated with germline *TP53* mutations (22, 23, 80–82).

The correlation between TP53 mutation (both somatic and germline) and MB molecular subgroup has been investigated. In 2013, Zhukowa et al. analyzed a cohort of 397 individuals affected by MB (age 1.1 to 45 years) and reported a *TP53* mutation almost exclusively in WNT and SHH subgroups while it was virtually absent in subgroups 3 and 4. They described a high difference in age distribution between MB_SHH_/*TP53* mutated, which are almost exclusively between ages 5 and 18 years, and MB_SHH_/*TP53* wild-type, that showed a bimodal distribution with peaks before 9 and after 18 years of age. Another interesting fact was that all individuals with *TP53* germline mutation, therefore affected by LFS, had MB_SHH_, and no germline mutations were observed in MB_WNT_/*TP53* mutated. For individuals with *TP53* mutant tumors, a dramatic association between biologic subgroups and survival was observed. Patients with MB_SHH_/*TP53* mutated showed a lower 5-year OS than those MB_SHH_ without *TP53* alteration (41% +/- 9% vs 81% +/- 5% respectively); on the contrary, individuals with MB_WNT_/*TP53* mutated showed an almost similar 5-year OS than those MB_WNT_ without *TP53* alteration (90% +/- 9% vs 97% +/- 3% respectively), demonstrating that *TP53* mutation status is much more crucial in the SHH subgroup. Within the limitation of the small cohort, no significant difference was observed between LFS children with MB_SHH_ and MB_SHH_ with somatic mutations of *TP53* (25).

### Diagnosis

The original definition of LFS requires one individual with a sarcoma diagnosed under the age of 45 that has at least one first-degree relative (parent, sibling, or child) with a cancer of any kind diagnosed under the age of 45 and a third family member who is either a first- or second-degree relative in the same parental lineage (grandparent, aunt, uncle, niece, nephew, or grandchild) with any cancer diagnosed under the age of 45, or a sarcoma at any age (83, 84). The finding of *TP53* mutations that did not fully respect classical criteria for LFS diagnosis led to the formulation of revised Chompret criteria. Individuals who meet classic and/or revised Chompret diagnostic criteria (**Appendix A**) should undergo TP53 genetic testing (65, 68, 71, 85).

### Cancer Surveillance

Cancer screening in LFS individuals is challenging due to the wide range of associated tumors. Villani et al. in a prospective observational follow-up study of a comprehensive clinical surveillance protocol identified 89 carriers of *TP53* pathogenic variants in 39 unrelated families and divided them in two groups: carriers who accepted surveillance (45%) and carriers who did not accept (55%); 21% of patients crossed over from the non-surveillance to the surveillance group for a total of 66% patients undergoing surveillance for a median of 32 months (86). Over an 11-year period, they identified 40 asymptomatic tumors in 32% of individuals who underwent surveillance and 60 symptomatic neoplasms in 88% patients who initially declined surveillance. The authors highlighted a significant survival advantage in individuals who underwent surveillance reporting 5-year OS of 88.8% in patients with the surveillance group and 59.6% in patients in the non-surveillance group. The Villani et al. 2016 version of the surveillance protocol for children with germline *TP53* pathogenic variants is summarized in **Table 6** (86). According to Ballinger et al. baseline whole-body magnetic resonance imaging can be used to identify early tumors in a highly cancer-prone population such as LFS patients, although further studies are needed (87).

**Table 6:** Villani et al. 2016 version of the surveillance protocol for children (birth to age 18 years) with germline *TP53* pathogenic variants.

**Adrenocortical Carcinoma**	Ultrasound of abdomen and pelvis every 3–4 months Blood tests every 3–4 months: 17-OH-progesterone, total testosterone, dehydroepiandrosterone sulfate, androstenedione 24 h urine cortisol, if feasible
**Brain tumor**	Annual brain MRI
**Soft tissue and bone sarcoma**	Annual rapid whole-body MRI
**Leukemia or lymphoma**	Blood tests every 3–4 months*: complete blood count, erythrocyte sedimentation rate, lactate dehydrogenase
**General assessment**	Complete physical examination every 3–4 months, including anthropometric measurements plotted on a growth curve (with particular attention to rapid acceleration in weight or height), signs of virilization (pubic hair, axillary moisture, adult body odor, androgenic hair loss, clitoromegaly, or penile growth) and full neurological assessment Prompt assessment with primary care physician for any medical concerns

*Serial specimens obtained at the same time of day and processed in the same laboratory.

### Therapeutic Approaches

Currently, there is no targetable therapy against tumors of LFS patients available. Generally, it is recommended to avoid use of DNA-damaging agents such as ionizing radiation in order to reduce the risk of secondary tumors with the exception of high grade CNS tumors. Notably, CNS tumor patients with LFS tend to show an overall worse outcome when compared to patients with the same CNS tumors but without TP53 alteration (78, 88, 89). Even though no guidelines exist, LFS patients should be subjected to physical examination annually with particular attention to neurologic functions. Radiologic approaches without ionizing radiation such as whole-body MRI are currently under investigation (81, 86).

## Turcot Syndrome

Turcot syndrome (TS) is defined by the association of colorectal cancer (CRC) and primary brain tumors and is one of the clinical manifestations of the mismatch repair cancer syndrome (OMIM # 276300). The first clinical report of the association of primary brain tumor and colorectal polyposis dates back to 1949 by Crail et al. (90). Ten years later Jacques Turcot described two siblings both affected by adenomatous colorectal polyposis and a malignant tumor of CNS, suggesting a common origin for this association (91). Two types of TS are known in literature. Type 1 (TS1) is characterized by the association between hereditary non-polyposis colorectal cancer (HNPCC), also called Lynch syndrome (LS), genetically related to the mutation of the mismatch repair (MMR) genes and CNS tumor (most frequently glioma). Type 2 (TS2) is characterized by the association of brain tumor and colorectal cancer due to familial adenomatous polyposis (FAP), caused by the mutation of the adenomatous polyposis coli (*APC*) gene, a suppressor gene in the long arm of chromosome 5 (92). Up to 10% of all CRC are inherited and among them a small number, commonly HNPCC or FAP, would be TS (93). Brain tumors in TS are mainly glioblastomas, associated with *MMR* genes mutations (TS1), and MB, associated with *APC* gene mutations (TS2).

### Turcot Syndrome Type 1

#### Genetic Basis

There is a strong association between TS1 and LS. Lynch syndrome is caused by heterozygous germline mutations, inherited in an autosomal-dominant manner, in any of the MMR genes (*MLH1*; *MSH2*, *MSH6*, *PMS1*; *PMS2*), which are involved in DNA repair pathway. Unlike LS, TS1 is caused by homozygous mutations in the aforementioned genes (94, 95).

#### Clinical Phenotype

TS1 can clinically manifest with both gastrointestinal (diarrhea, constipation, and/or a positive fecal occult blood test) and neurological symptoms depending on which tumor arises first (95). Lynch syndrome is characterized by an average age of onset that is earlier than in sporadic cases (45 vs 63 years) and by CRC that develops most frequently proximal to splenic flexure and can often be synchronous and metachronous (94). Regarding the development of extracolonic cancers the most frequent are represented by carcinoma of the endometrium, ovary, stomach, small bowel, pancreas, hepatobiliary tract, brain, upper uroepithelial tract, sebaceous adenomas and carcinomas, and multiple keratoacanthomas (94). TS1 patients may have skin signs such as café-au-lait spots, resembling type 1 neurofibromatosis, which instead are not reported in TS2 patients (95).

#### Correlation With Medulloblastoma

MB cases within TS1 are less frequently described than those reported in the setting of TS2, while gliomas are the most frequently reported brain tumors in TS1 (96–99).

In 2007, Scott et al. described a 13-year-old girl with two colonic carcinomas and MB diagnosed at the age of 7 years caused by constitutional biallelic mutations in the mismatch repair gene *MSH6*, the first case of MB reported in literature that was caused by the aforementioned biallelic alteration (100). Another report by Lindsay et al. described a 12-year-old with colonic adenocarcinoma and classic MB due to biallelic deletion in *PMS2* gene (101). To our knowledge, a correlation between TS1 and various subgroups of MB has not yet been highlighted.

#### Diagnosis

Some aspects should be considered in TS1 diagnosis: individuals with TS1 are offspring of consanguineous in 20% of cases, with no family history of brain tumors or colon; in TS1 polyps are larger and less numerous than in TS2; in TS1 skin lesions are café-au-lait spots while in TS2 they resemble epidermal cysts (95).

According to the American College of Gastroenterology all newly diagnosed CRCs should be studied for MMR deficiency with immunohistochemical testing for the MLH1, MSH2, MSH6, PMS2 proteins and/or with testing for microsatellite instability. Individuals with a history of a tumor that is suspected to be determined by MMR deficiency, a known family mutation associated with LS, or a risk ≥5% of LS obtained with risk prediction models should undergo genetic testing: discovering LS may sometimes be the first step toward diagnosing TS1 (102).

#### Cancer Surveillance

Cancer surveillance guidelines for patients at risk of or affected by LS have been published while, to our knowledge, no specific guidelines regarding the brain tumor surveillance in patients with TS1 have been established (102).

#### Therapeutic Approaches

Immunotherapeutic agents such as checkpoint inhibitors have been used in children with biallelic MMR deficiency glioblastoma multiforme, with encouraging results in some studies (26, 103). Checkpoint inhibitors seems to be effective in patients whose tumors harbor a high mutation load, resulting in the expression of neoantigens that act as a target for immunotherapy. Checkpoint inhibitors, through different mechanisms, activate T cells that recognize cancer cells as foreign by destroying them. Nivolumab is an anti-programmed death-1 (PD-1) directed checkpoint inhibitor, approved for the treatment of non-small-cell lung cancer and melanoma, and is being tested in various adult and pediatric tumors (103). Ipilimumab is an anti-cytotoxic T lymphocyte-associated protein 4 (CTLA-4) approved for the treatment of advanced melanoma and renal cell carcinoma and is also under clinical investigation in multiple adult and pediatric cancers (26). To our knowledge, there are no studies that have demonstrated the effectiveness of checkpoint inhibitors in children with MB, and therefore in those associated with MMR deficiency. Nivolumab and Ipilimumab are currently under investigation in a phase II trial of pediatric patients with high-grade CNS malignancies, including medulloblastoma (NCT03130959) (104).

### Turcot Syndrome Type 2

#### Genetic Basis

*APC* mutation is generally inherited with an autosomal dominant manner for the development of FAP, while TS2 seems to require a biallelic loss of the *APC* gene (92, 105). Indeed, in patients with a germ-line alteration of *APC*, inactivation of the second copy of the gene seems to be crucial for brain tumor development.

#### Clinical Phenotype

Clinical findings are those typically associated with colorectal cancer and brain tumors, which can occur at different times. Patients with TS2 tend to develop a number of polyps, around thousands, and they frequently manifest gastrointestinal symptoms (similar to those mentioned for TS1). Either before or after the polyps are found, various neurological symptoms and signs can arise, depending on the location of the tumor: headache, vomiting, visual and/or hearing problems, and sensorimotor deficits. In TS2 patients brain tumors can occur without polyposis, and this could be explained by the hypothesis that affected individuals die before adenomatous polyps have time to develop. Skin lesions can also occur in patients with TS2 and are most commonly epidermal cysts.

#### Correlation With Medulloblastoma

About 40% of patients with TS develop MB (95). According to Hamilton et al., the relative risk of MB in patients with FAP was 92 times higher than in the general population (92). Surun et al. in their multicentric retrospective review of 12 patients, treated between 1988 and 2018 for MB with an identified or highly suspected *APC* germline pathogenic variant, described some recurrent features such as a constant classic histopathology, a frequent lateral location, and a predominant nonmetastatic status. They highlighted a strong correlation between *APC*-mutated MB and WNT subgroup, demonstrating their excellent outcome, as indeed have wild-type-MB_WNT_ (106). An international multicenter study by Waszak et al., which included 1022 patients with MB, highlighted a close association between *APC* germline mutations and WNT subgroup; in this study germline *APC* mutations were found in five (71%) of seven *CTNNB1*-wild type MB_WNT_ cases, representing 7.6% of all MB_WNT_, which together with the counterpart constituted by somatic mutations of *CTNNB1* (89.4%), account for 97% of all MB_WNT_ (22).

#### Diagnosis

A key point in the diagnosis of TS2 patients is represented by family history. Individuals who have one or both parents with CRC diagnosed at an early age should be monitored for pre-cancerous colorectal polyps. According to the American College of Gastroenterology an individual with a history of ≥ 10 colorectal adenomatous polyps, or suggestive extracolonic manifestations, without a family history of an underlying pathogenic mutation, should be referred for genetic testing. In addition, the referral for genetic testing is also indicated for relatives of an individual with a known pathogenic mutation in order to establish the presence or absence of that specific mutation and to understand whether the relatives should be considered at-risk subjects (102).

#### Cancer Surveillance

The identification of family history of FAP and/or APC gene mutations may allow the clinician to perform surveillance in order to promptly identify the possible appearance of a brain tumor. An early diagnosis can allow an earlier treatment. However, it seems there is no advantage in terms of cost-effectiveness since not all individuals who present a CRC at an early age then develop a brain tumor and inversely (27, 95). Cancer surveillance guidelines for patients with FAP have been published, while, to our knowledge, no specific guidelines regarding brain tumor surveillance in patients with TS2 have been established (95, 102, 107).

#### Therapeutic Approaches

There is currently no targeted therapy available against tumors arising in the setting of TS2.

## Recently Identified Genetic Syndromes Associated With Medulloblastoma Predisposition

Pathogenic germline mutations in *BRCA2*, *PALB2*, *GPR161*, and *ELP* genes have been recently associated with an increased risk of developing MB.

### Germline BRCA2 and PALB2 Mutations

The international multicenter study by Waszak et al. identified germline *BRCA2* mutations in 11 (1%) of 1022 patients with MB, 10 children and one adult, with a median age at diagnosis of 5.7 years (22). They observed compound heterozygosity at *BRCA2* in 4 (36%) of 11 patients, of which all developed MB_SHH_ and showed a worse Progression-Free Survival (PFS) and OS (25% at 5 years, respectively) compared to patients with heterozygous germline *BRCA2* mutations, which instead showed a 100% OS and PFS, without secondary neoplasms. Germline mutations in *BRCA2*, compared with 53105 controls, were associated with increased risk of MB_SHH_ and MB_Group3/4_ (22).

*BRCA2* biallelic mutations are known to be responsible for **Fanconi Anemia (FA)**. The association of FA with MB has been described in literature (108). FA is a syndrome characterized by a chromosomal instability associated with congenital anomalies, bone marrow failure, and an increased risk of developing acute myeloid leukemia, myelodysplastic syndrome, and a number of solid tumors. It is a genetically and phenotypically heterogeneous disorder, inherited with an autosomal recessive pattern (rarely X-linked). We reported a novel *BRCA2* mutation (c.2944_2944delA.) in a 35-month-old female with FA and diagnosis of two distinct MBs that had been previously treated for a nephroblastoma at the age of 15 months. Genetic testing on the patient’s DNA extracted from both peripheral blood and MB cells revealed the presence of compound heterozygosis for BRCA2 frameshift mutations. Molecular analysis showed a MB_SHH_ for both the first- and the second-diagnosed MB. However differences in localization, more aggressive histology, and distinct gene expression pattern led to hypothesize a second distinct tumor rather than a distant relapse from the first one (109). The identification of SHH subgroup in FA patients may play a crucial role for their treatment with the use of targeted therapies, especially in these individuals extremely sensitive to conventional treatments.

In 2016 we described a case report of a 7-year-old girl with a classic histotype MB_WNT_ and whose family history was negative for cancer (28). After six years of complete remission from MB the patient developed a secondary glioblastoma. Genetic testing for cancer predisposition syndromes was performed despite a negative family history for neoplasms, and we identified a maternal inherited heterozygous germline *BRCA2* mutation, an unusual finding, since cases described in literature were non-WNT subgroups and, to our knowledge, this was the first case of *BRCA2*-mutated MB_WNT_ reported so far.

Waszak et al. also reported pathogenic heterozygous germline *PALB2* mutations in five (<1%) of 1022 patients with MB, of which there were 3 with MB_SHH_, 1 with MB_Group3_, and 1 with MB_Group4_. Five-year OS and PFS for patients with germline *PALB2* mutations was 75% (22).

Interestingly, a correlation was described between germline BRCA2 and PALB2 mutations and homologous recombination repair deficiency (HRD)-like mutation spectrum, specifically for pediatric MB_SHH_ (89% of cases), revealing HRD as potential biomarker for cancer predisposition in this subgroup (22). Furthermore, the association between germline *BRCA2* and *PALB2* with HRD-like mutation spectrum can be exploited to evaluate the susceptibility to combination therapies with PARP inhibitors.

### GPR161 Mutations

Germline G protein-coupled receptor 161 (*GPR161*) mutations have recently been described by Begemann et al. as variants predisposing to pediatric MB (110). *GPR161* is located on chromosome 1q24.2 and is involved in various aspects of embryonic development, including granule cell proliferation (111, 112). Proliferation of granule cells in cerebellum is regulated by SHH ligand and becomes abnormal when SHH-signaling pathway is constitutively activated. GPR161 acts as a SHH-pathway suppressor and its loss of function causes MB development (113). The frequency of germline *GPR161* mutations in the general population is about 6 in 10,000 individuals (110)*. GPR161* biallelic inactivation, most frequently by copy-neutral loss of heterozygosity of chromosome 1q in individuals with heterozygous germline mutation, in the absence of other driver somatic events, has been associated with early TP53-wild-type-MB_SHH_ development (110). According to Begemann et al., overall prevalence of germline *GPR161* mutations among pediatric (age<18 years) and infant (age<4 years) patients with MB_SHH_ was 3.4% and 5.5%, respectively (110). Copy-neutral loss of heterozygosity of chromosome 1q was never reported in *GPR161* wild-type MB_SHH_; therefore, it can be considered a molecular feature (110).

### Germline ELP1 Mutations

Germline loss of function (LOF) variants in *ELP1* have recently been identified in strong association with MB**in pediatric age (114). ELP1 is a molecule that is part of the Elongator Complex, involved in epitranscriptomic tRNA modifications, whose main function is to modify wobble base uridines in the anticodon loop of tRNAs in order to ensure a correct translational elongation (29, 115–117). The loss of even a single subunit causes the dysregulation of the Elongator Complex with consequent proteome instability. The cerebellum is described as the site of greatest ELP1 expression during brain development (118, 119). According to Waszak et al., three consecutive mutational events are probably required for the development of *ELP1*-associated MB_SHH:_ a heterozygous germline *ELP1*LOF variant; somatic biallelic inactivation of *ELP1* with monoallelic inactivation of PTCH1 via loss of chromosome arm 9q and biallelic inactivation of the residual PTCH1 allele via a somatic mutation or focal deletion (114). Interestingly, Waszak et al. found a strong association between germline LOF variants in *ELP1* and MB_SHH_ subgroup, especially with SHHα subtype (114). Patients with *ELP1*-associated MB_SHH_ showed a median age at diagnosis of 6.3 years, older than patients with MB_SHH_ and germline SUFU or PTCH1 LOF variants and younger than those with MB_SHH_ and germline TP53 mutations. These patients most frequently presented a desmoplastic nodular histotype and showed a favorable clinical outcome with 92% 5-year OS (114).

Rubinstein-Taybi syndrome (RSTS) is an extremely rare genetic disease, with an incidence of 1 in 100,000 to 125,000 live births, characterized by intellectual disability, unusual behavior, postnatal growth retardation, and multiple congenital anomalies, most frequently of the face and distal limbs (120, 121). RSTS is caused by a heterozygous mutation in *cyclic-AMP regulated enhancer binding protein* (*CREBBP*) gene, a transcriptional co-activator gene on chromosome 16p13.3, in about 60% of affected individuals (122), a submicroscopic deletion on chromosome 16p13.3 in about 10% of individuals (RSTS1, OMIM #180849) (123), alteration of *E1A binding protein* *p300 (EP300)* on chromosome 22q13.2 in about 5–10% of individuals (RSTS2, OMIM #613684) (124, 125). *CREBBP* gene and *EP300* genes act as transcriptional co-activators and are involved in DNA repair, cellular growth, differentiation, apoptosis, and tumor suppression (126). According to Boot et al. that reviewed the literature from 1963 to 2017, a total of 132 tumors have been reported in 115 individuals with RSTS and MB was the second most frequent CNS neoplasm with 6 reported cases, after meningioma (121). However, an increased risk for malignant tumors in RSTS could not be confirmed given the small numbers of affected individuals reported in literature, and additional studies are warranted.

## Genetic Testing of Cancer Predisposition Syndromes

With the advent of next generation sequencing (NGS) and implementation of genetic testing for adult cancer predisposition syndromes into routine clinical practice, cancer genetics research has extended the use of molecular testing for tumor and germline analysis in pediatric cancer patients. Molecular diagnosis of cancer predisposition syndromes can influence cancer screening initiation or frequency, to either prevent or detect cancer at an earlier and more treatable stage, and directly impact treatment decisions. However, even if medulloblastoma can be associated with rare hereditary cancer predisposition syndromes, screening guidelines for genetic counseling and testing of pediatric patients are not available (23). For genetic testing of cancer predisposition syndromes, different approaches are being used, and, currently, most molecular diagnostics laboratories that offer NGS are performing targeted gene panel testing or clinical whole exome sequencing (WES), more rarely whole genome sequencing (WGS). A multi-gene panel usually includes high and moderate penetrance genes and, sometimes, some low or of yet unknown risk genes, offering the advantage of identifying germline pathogenic variants in genes that would normally not be tested based on the patient’s diagnosis. However, it is possible that variants in genes not included in the panels contribute to the cancer risk and WES or WGS can be used to explore other genetic basis of familial syndromes in a more extensive way, permitting to identify new high- and moderate-risk genes of cancer predisposition. Genome-wide approaches generate huge amounts of genetic data and it remains a challenge to interpret the identified variants. Such data interpretation needs close collaboration among molecular geneticists, bioinformaticians, and clinicians. However, as sequencing costs are decreasing and computer and technological resources are expanding, genome-wide analysis in clinical practice will become more common.

## Conclusions

MB is the most frequent malignant CNS tumor in children, and additionally to the sporadic form, MB can occur in association with a cancer predisposition syndrome. Knowledge of the clinical findings, etiopathogenic basis, and diagnostic criteria of each syndrome described in this review allow the pediatrician to make a correct diagnosis, start cancer surveillance, and suspect precociously a MB on its onset, providing a prompt treatment. Conversely, when MB is diagnosed, the correct identification/detection of a cancer predisposition syndrome can allow the clinician to make a more appropriate and complete management of treatment involving several medical specialists in a multidisciplinary team. The molecular studies conducted in the last years have evidenced an association between the various cancer predisposition syndromes and the different MB subgroups. Knowing these relationships can help further clarify the difference not only from a biological point of view but also in prognostic terms. Notably, the extremely poor outcome of MB_SHH_ in children expressing germline *TP53* mutations has already been reported. Based on the findings described by Waszak et al., pediatric MB_SHH_ development could be explained by a high genetic predisposition (about 40%); therefore, the effort to carry out genetic testing and surveillance program for affected patients and families in this subgroup becomes even more crucial.

According to Waszak et al. we suggest that patients with MB_SHH_ should be tested for germline *TP53* (when older than 3 years), *SUFU* and *PTCH1* mutations (when younger than 3 years), and if negative, also for germline mutations in *BRCA2* and *PALB2*. Furthermore, we suggest that patients with MB_SHH_ should be tested for germline *ELP*1 mutations, especially those presenting outside of infancy, and for germline *GPR161* mutations, particularly those presenting in infancy. We suggest, also, genetic counselling for germline *APC* mutations in children with MB_WNT_.

Considering that only 5–6% of MB are associated with cancer predisposition syndromes, our current knowledge is probably still limited. Given the importance that the recognition of a cancer predisposition syndrome can have in the management of a child with MB, we suggest to extend genetic testing also in patients with family history for cancer and/or finding of a dysmorphic phenotype. Knowledge of the associations between molecular subgroups and cancer predisposition syndromes can also be useful in clarifying the differences in terms of therapeutic vulnerability, guiding the development of new targeted therapies. Finally, the comprehension of these biological and molecular differences can help to further improve cancer surveillance measures, with the aim of guaranteeing the best quality of care for the patients.

## Author Contributions

RC and GB equally contributed to this manuscript. EM, AP, ZMB, FN, GC, EP, EA, MR, ML, and AC contributed to the finishing of the work. FN provided the figure. AM, ACar, EF, LB, and FL revised it critically for important intellectual content. All authors finally approved the version to be published and agreed to be accountable for all aspects of the work in ensuring that questions related to the accuracy or integrity of any part of the work are appropriately investigated and resolved. All authors contributed to the article and approved the submitted version.

## Conflict of Interest

The authors declare that the research was conducted in the absence of any commercial or financial relationships that could be construed as a potential conflict of interest.

## Appendix A. Li-Fraumeni syndrome classic diagnostic criteria and revised Chompret criteria

**Classic diagnostic criteria**

A proband with Sarcoma diagnosed under the age of 45 years

**AND**

A first degree relative with any cancer under 45 years

**AND**

Another first or second degree relative with either cancer under 45 years or a sarcoma at any age

**Chompret diagnostic criteria (revised)**

A proband with an LFS spectrum tumor (soft tissue sarcoma, osteosarcoma, brain tumors, pre-menopausal breast cancer, adrenal cortical carcinoma, leukemia, lung bronchoalveolar cancer) before 46 years

**AND one of the following criteria:**

At least one first- or second-degree relative with an LFS tumor (except breast cancer, if the proband has breast cancer) before 56 years or with multiple primary tumors

**OR**

A proband with multiple primary tumors (except multiple breast tumors), two of which belong to the LFS tumor spectrum and the first of which occurred before 46 years

**OR**

A proband with adrenal cortical carcinoma or choroid plexus carcinoma or embryonal anaplastic subtype rhabdomyosarcoma independent of the family history

**OR**

Breast cancer before the age of 31 years
