# Supplementary material for: Cancer Predisposition Syndromes and Medulloblastoma in the Molecular Era
Source: Front Oncol. 2020 Oct 29;10:566822. doi: 10.3389/fonc.2020.566822 (PMC7658916; doi:10.3389/fonc.2020.566822)
Supplement: Supplementary file 1 [file DataSheet_1.docx]

**Appendix A. Li-Fraumeni syndrome classic diagnostic criteria and revised Chompret criteria**

**Classic diagnostic criteria**

A proband with Sarcoma diagnosed under the age of 45 years

**AND**

A first degree relative with any cancer under 45 years

**AND**

Another first or second degree relative with either cancer under 45 years or a sarcoma at any age

**Chompret diagnostic criteria (revised)**

A proband with an LFS spectrum tumor (soft tissue sarcoma, osteosarcoma, brain tumors, pre-menopausal breast cancer, adrenal cortical carcinoma, leukaemia, lung bronchoalveolar cancer) before 46 years

**AND one of the following criteria:**

At least one first- or second-degree relative with an LFS tumor (except breast cancer, if the proband has breast cancer) before 56 years or with multiple primary tumors

**OR**

A proband with multiple primary tumors (except multiple breast tumors), two of which belong to the LFS tumor spectrum and the first of which occurred before 46 years

**OR**

A proband with adrenal cortical carcinoma or choroid plexus carcinoma or embryonal anaplastic subtype rhabdomyosarcoma independent of the family history

**OR**

Breast cancer before the age of 31 years
